# The development of complex and controversial innovations. Genetically modified mosquitoes for malaria eradication

**DOI:** 10.1016/j.respol.2019.103917

**Published:** 2020-04

**Authors:** Valentina Cisnetto, James Barlow

**Affiliations:** a Imperial College London, Department of Life Sciences, South Kensington Campus, London, SW7 2AZ, United Kingdom; b Imperial College Business School, South Kensington Campus, London, SW7 2AZ, United Kingdom

**Keywords:** New product development, Adoption, Genetically modified mosquitoes, Living technology, Gene drive, Malaria

## Abstract

When there is significant uncertainty in an innovation project, research literature suggests that strictly sequencing actions and stages may not be an appropriate mode of project management. We use a longitudinal process approach and qualitative system dynamics modelling to study the development of genetically modified (GM) mosquitoes for malaria eradication in an African country. Our data were collected in real time, from early scientific research to deployment of the first prototype mosquitoes in the field. The 'gene drive' technology for modifying the mosquitoes is highly complex and controversial due to risks associated with its characteristics as a living, self-replicating technology. We show that in this case the innovation journey is linear and highly structured, but also embedded within a wider system of adoption that displays emergent behaviour. Although the need to control risks associated with the technology imposes a linearity to the NPD process, there are possibilities for deviation from a more structured sequence of stages. This arises from the effects of feedback loops in the wider system of evidence creation and learning at the population and governance levels, which cumulatively impact on acceptance of the innovation. The NPD and adoption processes are therefore closely intertwined, meaning that the endpoint for R&D and beginning of 'mainstream' adoption and diffusion are unclear. A key challenge for those responsible for NPD and its regulation is to plan for the adoption of the technology while simultaneously conducting its scientific and technical development.

## Highlights

• Using the example of mosquitoes that are genetically modified for malaria eradication through gene drive methods, a scientifically complex ‘living technology’, we show how complexity, uncertainty and risk can propel NPD processes towards a linear sequence of stages.
• Although the need to control risks associated with gene drive technology imposes linearity to the NPD process, there are possibilities for deviation from a structured sequence of stages. This is due to the effects of feedback loops in the wider system of evidence creation and learning at the population and governance levels, which cumulatively impact on acceptance of the innovation.
• The NPD and adoption processes involved in the use of gene drive technology are closely intertwined, and the endpoint for R&D and beginning of ‘mainstream’ adoption and diffusion are unclear.

## 1 Introduction

Innovation journeys of complex innovations are seen as unpredictable and non-linear, shaped by the context of organizations and institutions involved in NPD, adoption and implementation (Cheng et al., 1996; Van de Ven et al., 1999; Garud et al., 2013; Van de Ven, 2017). Emerging literature on management processes in complex projects has investigated valuation, decision making and project management (de Rezende et al., 2018; Brasil et al., 2018; Le Masson et al., 2019; van Oorscott et al., 2018). However, research on new product development (NPD) has generally avoided instances where there is significant complexity in either or both the product itself and its context for adoption (Pich et al., 2002; Sommer and Loch, 2009; Sihvonena and Pajunen, 2019). There have been longstanding calls for more longitudinal and multilevel research on NPD in cases of complex or large-scale innovations (Montoya-Weiss and Calantone, 1994; Klein and Sorra, 1996; Anderson et al., 2004; Gupta et al., 2007; Hitt et al., 2007; Page and Schirr, 2008; Crossan and Apaydin, 2010; Perks and Roberts, 2013; Hueske et al., 2015; Ardito et al., 2015; Van de Ven, 2017).

Also underemphasized in literature is the relationship between the processes of NPD and those of innovation adoption and implementation. A review of strategic management literature (Keupp et al., 2012) found only three out of 342 studies focused on implementation, despite repeated calls dating back to the 1990s for more research (Klein and Sorra, 1996; Repenning, 2002). The assumption tends to be that an innovation journey ends when a new product or service is launched (Anderson et al., 2004). An exception is research on the adoption of healthcare innovations, which recognises that implementation is often a far bigger challenge than NPD (Edmondson et al., 2001; Greenhalgh et al., 2004; May and Finch., 2009).

In this paper we explore the processes of NPD and adoption through the lens a highly complex innovation from the emerging and under-researched field of ‘living technology’, where biology plays an active part in the development process. Living technologies include bio-engineered drugs and foodstuffs, and genetically modified (GM) organisms; they are complex in their science and frequently controversial (Russell, 2008; Fischer, 2016; Wickson et al., 2016). While there is research on the impact of living technologies in agriculture (Raparelli et al., 2018) and medical genomics (Williams, 2018), literature is rather sparse on the management of its R&D processes, apart from work on economic and organizational models in the biopharmaceutical industry (Lim et al., 2006; Schuhmacher et al., 2016; de Villemeur et al., 2019). By studying the development of a novel health innovation – the application of ‘gene drive’ technology to modify malaria-carrying mosquitoes – from its scientific research work to release of the first version of modified mosquitoes we provide insight into R&D management in living technology, and consequently lessons for the study of NPD processes in complex, large-scale technologies more generally.

## 2 Understanding new product development

New product development (NPD) is the set of processes by which new ideas or technologies are materialized, managed and eventually brought to market (Mu et al., 2009). It can be fraught with technological, marketing and organizational risks, often influenced by the external and internal environment in which NPD takes place (Doering and Parayre, 2000). Complexity in the product itself or its wider environment can make it hard for firms to understand and accurately predict the actions of rivals or the extent to which the innovation will function as promised (Ragatz et al., 2002; Ignatius et al., 2012; Mu et al., 2009; Beck da Silva Etges et al., 2019; Hullova et al., 2019a). In the early stages of NPD and for new-to-the-world innovations, there may be a high degree of uncertainty (the difference between information required to perform a particular task and amount of information already possessed), equivocality (the assumption that in a socially constructed world, meanings need to be made sense of and negotiated) and complexity (the non-linear interaction between parts of a system) (Stevens, 2014; Reid and De Brentani, 2004). New-to-the-world innovation fundamentally involves uncertainty in which the set of possible options and their outcomes may be unknown and unforeseeable (Dequech, 2011).

Complexity in NPD arises in a number of ways. It is often associated with the scale of the project – the number of technologies, components, organisations or business functions that are involved (Griffin, 1997; Nightingale, 2000; Kim and Wilemon, 2003; Dunne and Dougherty, 2016; Dougherty, 2017). Complexity may arise from NPD activities themselves – the number of different R&D tasks and their interdependence (Ahmad et al., 2013; Chapman and Hyland, 2004; Dooley and Van de Ven, 1999; McCarthy et al., 2006) or novelty of these tasks (Chao et al., 2014; Maggitti, et al., 2013; Tatikonda and Rosenthal, 2000). Others have suggested the importance of temporal and cultural factors (Oeija et al., 2019).

Various strategies for managing risks associated with complexity in NPD projects have been put forward. Typically, technology developers try to convert uncertainty into measurable risk using some form of structured decision-making process, with clear stages and rules to decide whether to proceed or abandon a project. These might start with an evaluation of market or user needs, and progress through technological R&D and prototyping, before product launch. A well-known approach is the ‘stage gate’ model (Cooper, 2008). However, when there is significant uncertainty or complexity, NPD processes are unlikely to be reducible to a step-by-step unfolding sequence of actions and stages (Anderson et al., 2004; Van de Ven, 2017). Simple stage-gate approaches are therefore seen as inflexible, unable to cope with serendipity that often arises during NPD and the complexities of the competitive or regulatory environments (Salerno et al., 2015; Hullova et al., 2019a), and based on misleading assumptions about the origin and development of innovations (Van de Ven et al., 1999; Van de Ven, 2017). Alternative strategies focus on reducing project interdependencies (Mihm et al., 2003) or supporting complementary rather than conflicting dependencies (Oyama et al. 2015). Others have suggested that complexity can be managed through hybrid approaches that combine elements of stage-gate models and more iterative approaches, breaking down complex processes into smaller components for problem solving (Iansiti and MacCormack, 1997; Nightingale, 2000; Sommer et al., 2015; Cooper, 2016; Sihvonena and Pajunen, 2019). Another option involves experimentation, where multiple variants of an innovation are tested in increasingly real-world situations (Matson, 1991; Thomke et al., 1998; Thomke, 2003), enabling rapid iterative learning from small-scale trials that allow risks to be contained and opportunities to be exposed faster. Some have questioned the relevance of this approach in situations of high uncertainty, where there is a lack of clarity on the scope of problems, testable alternatives and evaluation criteria (Packard et al., 2017; Gillier and Lenfle, 2019; Garud and Van de Ven, 1992; Van de Ven and Polley, 1992; Lynn et al., 1996; Sommer et al., 2009).

The variety of factors that underpin complexity, and range of strategies for its management, imply that NPD processes and innovation journeys in situations of complexity may be “neither stable and predictable nor stochastic and random” (Van de Ven et al., 1999: 5). Innovators will engage in non-linear and iterative cycles of development activities that over time eventually converge on more orderly patterns of behaviour, ultimately resulting in a new product or service (Cheng and Van de Ven, 1996; Van de Ven et al., 1999; Van de Ven, 2017). This process will be sensitive to the initial conditions in the overall system of organizations, markets and institutions involved in NPD and adoption, as well as unpredictable exogenous events. Consequently, there have been calls for perspectives that draw on complexity science to better understand NPD projects and their evolutionary nature (Anderson, 1999; McCarthy et al., 2006; Baumann and Siggelkow, 2013; Akgün et al., 2014; Oyama et al., 2015). This is seen as a way of introducing a temporal and multi-level perspective (Langley, et al., 2013), linking processes and outcomes at individual, organizational and macro levels in innovation projects (Klein and Sorra, 1996; Anderson et al., 2004; Gupta et al., 2007; Crossan and Apaydin, 2010; Hueske et al., 2015).

In summary, while there is a reasonable understanding at an aggregate level of the causes and implications of complexity, scholars have long noted a need for more empirical research on specific cases of complex NPD activities and their management (Pich et al., 2002; Sommer and Loch, 2009; Sihvonena and Pajunen, 2019). Studying NPD in the health technology sector could help to address these calls. Health technology innovation often involves a high degree of technological, organizational and institutional complexity. Hence, innovations need to be adapted to their local context, often following a sequence of experimentation and evidence-gathering in trials, since what works in one setting may not work elsewhere (Denis et al., 2002; Heath et al., 2003; Dopson, 2005; Ferlie et al., 2005). We now describe the basic features of health technology NPD, focusing on pharmaceutical and living technology (biopharmaceutical) innovations.

There are differences between the medical devices and pharmaceutical sectors in the way NPD, adoption and implementation occur; ‘living technologies’ represent a further category of health technology (Lehoux et al., 2014). For medical devices, diversity in products and technologies means that NPD processes vary, often influenced by the availability of funding and the knowledge and networks of the innovators (Dixon et al., 2006). The extent to which NPD follows a typical stage-gate process depends on the level of risk – financial and safety – and whether there are regulatory requirements for evidence of safety and efficacy. The development of new drugs involves a highly regulated and formalised stage-gate process of evidence gathering, beginning with basic and applied research, and followed by phased trials before approval and market launch (Northrup et al. 2012). There may be further studies to monitor for safety issues that only become apparent after a period of time or when the user population is large enough. A variant of the standard drug development model, used for rare diseases or when rapid generation of evidence is needed during epidemics, is known as ‘adaptive licensing’. A principle of adaptive licensing is that there is no single point when a drug is proved safe and effective; uncertainty is reduced iteratively through continued learning and development once adoption has commenced (Eichler et al., 2014).

The NPD processes of living technologies differ significantly from ‘traditional’ chemistry-based pharmaceuticals (Lehoux et al., 2014). Biological molecules are more complex and difficult to specify than chemistry-based molecules, so biopharmaceutical manufacturing processes are less predictable than those of traditional drugs (Lim et al., 2006). Not only is regulation needed to address safety and efficacy, but the pharmacological stability of the end product must also be guaranteed (Lim et al., 2006; Pfeffer, 2012). A further characteristic of some living technologies is ‘biological embeddedness’. The production system lies ‘inside’ the organism itself, hence the product may be able to reproduce itself without the need for a manufacturing facility or laboratory. There have long been concerns over the implications of this feature in GM-created agricultural crops (Raparelli et al., 2018). Biological embeddedness makes this technology socially and politically controversial, requiring close regulation (Russell and Sparrow 2008). Unpredictability of outcomes also implies that the adoption journey may be influenced by local interactions between scientists’ intentions, public understanding of the technology, and its institutional and soci0-economic ecosystem for R&D, regulation, adoption and use (Russell, 2008). This narrative characterizes GM as an “industrial innovation that circumvents or outflanks nature; its performance disembodied and disembedded from the natural and social system in which it operates” (Schnurr, 2012). As we discuss below, this is a particular feature of products created through ‘gene drive’ technology, the subject of our case study. Fischer (2016) argues that the concept of biological embeddedness highlights the central role of biology as an active agent in NPD processes of living technology, but empirical research is needed to understand the implications and build theory (cf. Russell and Sparrow 2008; Fischer and Eriksson, 2016; Wickson et al., 2016; Catacora‑Vargas et al., 2018).

## 3 Genetically modified mosquitoes as a ‘living health technology’

Malaria remains a major global health challenge (Sachs and Malaney, 2002; Feachem et al., 2019). Genetic modification (GM) technologies which eradicate mosquito species that function as a vector for the malaria parasite offer a possible cost-effective innovation that provides area-wide equitable and sustainable protection (Windbichler et al., 2011; Nolan et al., 2011; Bhatt et al., 2015; Rabinovich et al., 2017; Kyrou et al., 2018). Compared to conventional technologies for malaria control and eradication, an advantage of GM mosquitoes is that no change in human behaviour is needed for adoption and diffusion, unlike the case of insecticide treated bednets or drugs (Windbichler et al., 2011). As well as malaria, GM technology is being developed for insects carrying other vector borne diseases such as dengue fever. When released, sterile male mosquitoes mate with female wild-type mosquitoes but the offspring will not mature to adulthood; this causes a decline in the mosquito population and lower incidence of the target disease.

A GM species of the mosquitoes carrying dengue fever, developed by Oxitec, achieved an 80–96% reduction in mosquito population in field trials (Phuc et al., 2007; Labbé et al., 2010; Harris et al., 2012; Dhang, 2014), and was adopted in some regions (Servick, 2016). Oxitec's mosquitoes involve a ‘single-generation’ GM technology where the modification is not passed on. This means new modified mosquitoes need to be produced and released regularly. Use of gene drive technology overcomes this limitation by creating a strain of mosquitoes that can pass on modified genes to a disproportionately high percentage of offspring. Over successive generations, this should lead to a progressive reduction in malaria carrying mosquitoes until there are too few remaining to sustain transmission of the disease. The technology is fundamentally different from earlier types of GM because it breaks the normal rules of gene inheritance by creating a self-sustaining population (Burt, 2003; Burt and Koufopanou, 2004).

The novel science and untried techniques have made GM mosquitoes controversial since inception. Oxitec's mosquitoes were criticised because of the implications for species extinction (Pugh, 2016) and concern that ineffective deployment may lead to the spread of dengue (GeneWatch, 2012). Anxiety about the environmental impact resulted in a 170,000 signature petition opposing a trial in Florida (Glenza, 2016). Gene drive technology is especially controversial because of its potentially irreversible nature (Patrão Neves and Druml, 2017; Callaway, 2018; Collins, 2018; Brossard et al., 2019) – ‘adoption’ of gene drive mosquitoes is seen as is all or nothing. The extent of concern over GM and gene drive are reflected in the stringency of regulations and public opinion. The complexity and novelty make the technology hard to explain, resulting in significant ‘cognitive distance’ – dissonance and rejection of presented evidence (Festinger, 1962) – between scientists, policy makers, regulators and the public. Scientific and technological development must proceed slowly and iteratively, both to meet the needs of research and regulatory requirements, and to provide evidence of safety and efficacy to reassure the public and policy makers. Gene drive technology therefore faces substantial challenges in securing public trust and acceptance (Feachem at al., 2019).

What do these features imply for understanding NPD processes of ‘living technologies’? We have seen how innovation journeys where there is significant uncertainty or complexity can involve iterative cycles of apparently chaotic activities which converge on the final product, rather than a simple linear sequence of activities (Cheng et al., 1996; Van de Ven et al., 1999; Garud et al., 2013; Van de Ven, 2017). There are examples where scientifically complex technologies such as novel drugs follow highly prescribed and staged development pathways. But in living technologies, where there is inherent unpredictability due to biological embeddedness and the science is cutting-edge, complex and controversial, development processes may be highly sensitive to the social, regulatory and political context as well as the environmental ecosystem into which the innovation is deployed – there is likely to be uncertainty about potential outcomes in the field.

## 4 Case study setting

The setting for our research is a project to use gene drive technology to develop a modified, self-sustaining mosquito, released in selected African countries (our case study is in Burkina Faso). Founded in 2005, Target Malaria is a not-for-profit research consortium which aims to eradicate malaria through the development of GM mosquitoes, with core funding from the Bill & Melinda Gates Foundation and the Open Philanthropy Project Fund. Technology developed by Target Malaria is made available to lower-income countries at zero cost or costs limited to those arising from growing and delivering the mosquitoes. Research findings are published in the public domain (Kyrou et al., 2018; Collins et al., 2018; Burt et al., 2018a, 2018b).

Target Malaria is an ideal case through which to investigate NPD processes in a living technology because the technology is inherently complex and controversial, and its development and implementation require closely coordinated activity between actors ranging from global and national regulatory bodies to populations in local field sites, over many years. Intermediate versions of the final product require trialling in the laboratory and field, progressively scaling-up trials before release in the wild. Key milestones are the development of (1) self-limiting sterile male mosquitoes, (2) mosquitoes with self-limiting male bias, and (3) mosquitoes with self-sustaining male bias or fertility gene knock-out (Sinkins and Gould, 2006). Each of these types of mosquito is a new innovation in its own right, with its own NPD and adoption journey. Only the final version has all the characteristics needed to trigger self-sustaining modification of a mosquito population (Burt et al., 2018a, 2018b). Each milestone needs to be validated through research studies and open field releases of the new mosquito version; each is subject to regulatory approval. In parallel, there needs to be progressively greater readiness by the local population to accept the innovation, and the creation of a distribution network capable of delivering the modified mosquitoes as the scale and scope of trials increases.

**Fig. 1:**
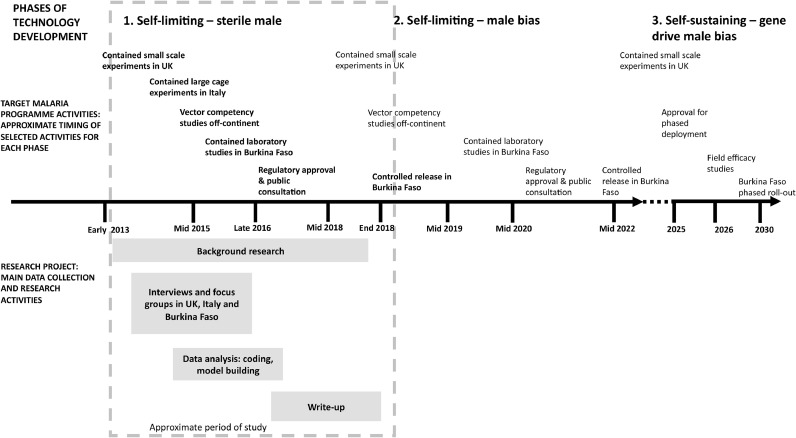
Target Malaria timeline.

We recognize that in an R&D project as long as Target Malaria our research conclusions may be influenced by the period over which our study is conducted (Pettigrew, 1990; Dubois and Gadde, 2002; Quintens and Matthyssens, 2010). We focus on a discrete six-year phase of Target Malaria (see Fig. 1), encompassing scientific research leading up to the creation of self-limiting sterile male mosquitoes and small-scale open field release. While this period is only approximately a third of the overall R&D programme, which should culminate in phased roll-out during the late 2020s, it nevertheless represents a distinct cycle of activity, with a definitive end point.

### 4.1 Research methods and data

We adopted a mixed methodology, combining interviews and focus groups with Target Malaria stakeholders (ranging from policy and regulatory bodies to local villagers) and an analytical strategy which drew on systems thinking and qualitative system dynamics modelling (Sterman, 2000). Our data collection comprised a range of activities over a five-year period: initial examination of Burkina Faso's socio-demographic, cultural, institutional, ecological and epidemiological context, exploratory interviews with key actors in Burkina Faso's health innovation ecosystem, local-level focus groups and semi-structured interviews in field sites, and finally meetings with key stakeholders to gather feedback on emerging findings. We aimed to identify the sequence of events and outcomes while they happened, in order to gain insight into decisions made about the NPD trajectory and identify potential causal relationships between the phenomena we observed.

Rather than collecting data during a limited number of points in time, we engaged continuously with Target Malaria. One author (VC) had previously worked as an associate project manager for Target Malaria. This ensured deeper engagement with the project teams and access to data which would have otherwise been hard to collect. She was able to regularly visit Burkina Faso, the Italian collaborating research facility and other research institutions involved in the project (we discuss the implications for research reflexivity in Section 8, limitations). Our fieldwork and other data collection, transcription and coding, discussion of preliminary explanatory categories and development of the system dynamics model were therefore evolving iteratively in parallel to events in the Target Malaria project. Emerging insights either stimulated further data collection or revision of our analytical model. This helped us to mitigate the problems of trying to rationalize events from data collected at only a few points in time (Macharzina and Engelhard, 1991; Hullova et al., 2019b).

**Fig. 2:**
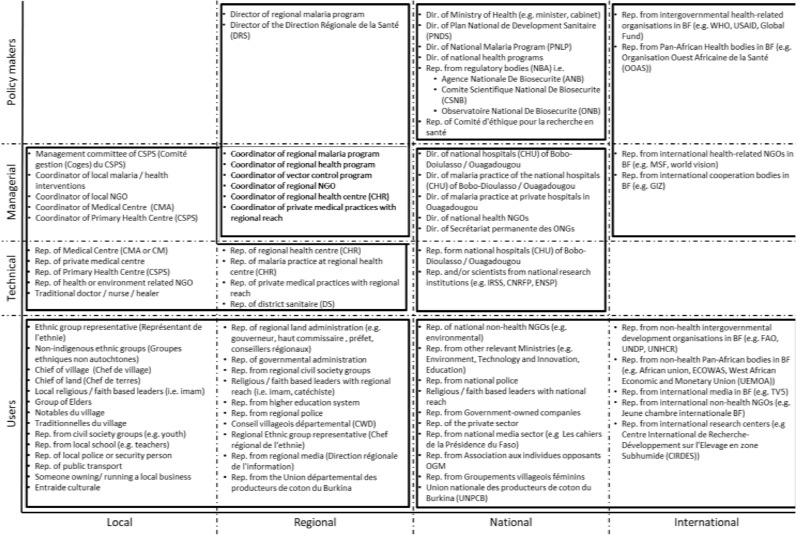
Stakeholder mapping with clusters for sample selection. Abbreviations: BF: Burkina Faso; CHR: Centres Hospitaliers Régional; CHU: Centres Hospitaliers Universitaires; CM: Centres Médicaux; CMA: Centres Médical avec Antenne Chirurgicale; CNRFP: Centre National de Recherche et de Formation sur le Paludisme; CSPS: Centres de Santé et de Promotion Sociale; Dir.: director; DRS: Direction Régionale de la Santé; ENSP: école nationale de santé publique; PNLP: Programme National de Lutte Contre le Paludisme; Rep.: representative.

We began our work with a preliminary examination of Burkina Faso's health system and socio-demographic context. This enabled us to identify key Burkinabé (Burkina Faso) individuals, institutions and organizations which shape the policy, regulatory and knowledge context for the introduction of GM mosquito technology. These were mapped and grouped into seven clusters, based on their geographical and policy level (local, regional, national, international) and their role in relation to the health and regulatory systems (see Fig. 2). Interviewees were purposively selected from these clusters to achieve a multi-level, broad-spectrum representation. This sample included government bodies, politicians, international organisations, NGOs, scientific communities, health sector workers and people from local communities in trial sites. Interviews were also carried out with Target Malaria's scientific, stakeholder engagement and regulatory teams from Burkina Faso and Europe. The list of stakeholders was enlarged through snowballing until data saturation was reached. Data also included documentary material (e.g. project reports) and observation of meetings and conference calls for Target Malaria working groups (product development, local capacity building, regulatory, risk assessment and management, communications, and stakeholder engagement). These data were coded and analysed, allowing triangulation with the interview and focus group data.

### 4.2 Interviews and focus groups

A semi-structured open-ended interview protocol was developed for each stakeholder cluster. We primarily asked representatives from the local and regional clusters about their perception of health and healthcare problems and the broad social and economic context. Interviews in the national policymaker cluster focused on characteristics and dynamics of the adoption and health systems. At the time of the interviews the Target Malaria project had just submitted an application to Burkina Faso's regulatory authority for contained use of sterile male mosquitoes. We were therefore unable to interview regulators about a technology they were currently reviewing. To gain an understanding of local regulatory issues we interviewed an expert on GM regulatory processes in Burkina Faso and another with experience of regulating mosquitoes created using different GM technologies.

One of the authors (VC) travelled to Burkina Faso six times during the study period to collect and validate the data. In total, 42 in-depth semi-structured interviews and three focus groups were conducted, with data collected from a total of 74 participants. At least one person per stakeholder cluster was interviewed. There was one refusal and one interviewee did not want to be recorded. Interviews in Burkina Faso were either conducted in the local language with a translator or in French, based on the interviewee's language fluency. Both authors have a good understanding of French.

**Fig. 3:**
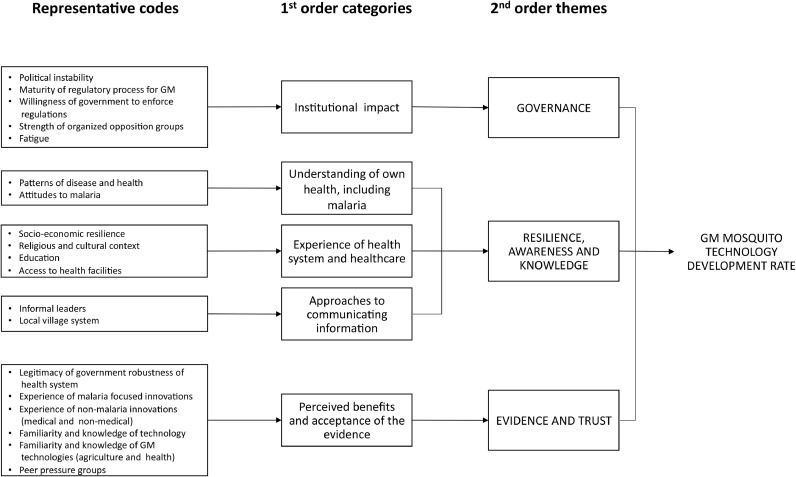
Data codes, categories and themes.

The collected data were transcribed and analysed, coded and clustered (Corbin and Strauss, 2008). We triangulated these data by comparing them with documentary material acquired during the field trips and from observation of Target Malaria project management activities. From an initial set of notes and preliminary codes, discussed and agreed by the authors, we began to identify similarities and relate the codes to each other through axial coding (Locke, 1996). The analysis was iterative in order to continuously explore and agree the codes and emerging themes. This helped us structure the data into five first-order categories from which to derive three broader themes (Gioia et al., 2012) (Fig. 3). We used NVivo 10 for analysis of the transcripts.

### 4.3 Qualitative system dynamics modelling

The codes, categories and themes helped indicate possible relationships, but not an explanation for the dynamics surrounding Target Malaria's NPD process. One author (JB) had experience of using qualitative system dynamics modelling to explore possible causality in the relationships and role of time lags within a complex innovation programme (Dattée and Barlow, 2017). This method enables a structured analysis of the relationships between components of a system (Craig et al., 2008; Shepperd et al., 2009), especially where contextual factors can lead to multiple outcomes within and outside the system (Jun et al., 1999; Guenal and Pidd, 2010). This is typical of health systems or complex technologies where there is uncertainty over how choices might affect final outcomes. Delays, feedback and non-linearities that influence the dynamics of the system can be included in these models. The approach therefore allows for better understanding of possible counter-intuitive behaviour and unintended consequences of interventions in systems at different scales of implementation and impact, and different timescales.

We describe the structure of the model in detail in Section 5. To build the model we followed Sterman (2000). We began by creating causal loop diagrams representing relationships within and between emerging themes from our data (cf. Azoulay et al., 2010; Dattée and Barlow, 2017). This process is useful for eliciting and capturing mental models and hypotheses about a system's behaviour, especially in relation to the endogenous and exogenous consequences of its feedback structure. Causal loops can take the form of ‘reinforcing loops’, which create positive feedback that enhances change in a certain direction. ‘Balancing loops’ resist further increases in a given direction, so that a system will try to return to a desired state and maintain itself there.

As we developed the model, we tried to ensure that its boundaries and the level of detail were kept in balance. It was important to avoid trying to build a model that ‘explains everything’ and to maintain our focus on the research objectives. This was essential to create a useful representation of the factors influencing the NPD process and to capture explanatory feedback loops, especially those that might be unaccounted for in the mental models of the stakeholders and authors.

Modelling often works best as an iterative process between modellers and stakeholders involved in the phenomenon being modelled (Sterman, 2000). Unfortunately, this was not possible due to the logistical difficulties in frequently engaging with stakeholders in Burkina Faso. However, validation of the model was achieved through discussion between the authors and the Target Malaria team, which helped to challenge its assumptions and build confidence in the model. The emerging model was also reviewed independently by three experts in system dynamics modelling in healthcare.

## 5 Analysis and findings

**Fig. 4:**
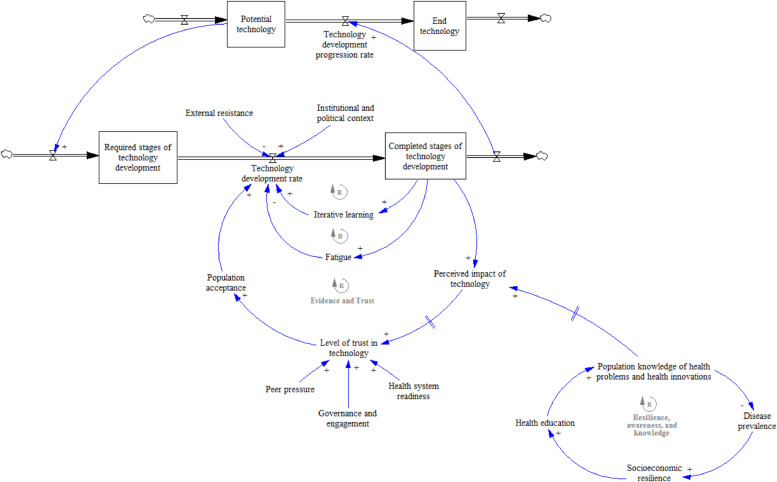
Overview of the system dynamics model of the development and adoption of GM mosquitoes in Burkina Faso. Reinforcing loops are marked by an R with an arrow in the direction of the loop. Balancing loops are marked by a B with an arrow in the direction of the loop.

As we analysed our data and began to develop the model it became clear that two groups of interacting factors influence the processes of developing and introducing GM mosquitoes in the Burkinabé context: the impact of policy and regulatory institutions (sub-model 1) and socio-economic factors influencing population acceptance (sub-model 2). Fig. 4 provides a high-level overview of the model describing these interactions. Next we briefly describe our overall model, before discussing the sub-models in more detail.

### 5.1 Model overview

System dynamics models involve causal loops describing the dynamics of a system and ‘stocks and flows’ which describe the state of the system. Fig. 4 shows that the NPD progression rate can be conceived as a stock and flow relationship between the completed stages of technology development and the remaining required stages, i.e. from original concept to fully developed self-sustaining GM mosquitoes. In other words, at any given time a certain proportion of the total effort needed to progress from a potential to an actual innovation deployed in the field will have been expended. The faster the product development rates in each of the stages, the faster the progression rate from potential to final technology. The stock and flow therefore capture the three stages of GM mosquito development, and testing and regulatory approval milestones (Section 4). Each stage entails R&D activity both within and outside Burkina Faso, deployment of mosquitoes in increasingly large-scale field studies, and regulatory and local community approval prior to further development.

The model incorporates the dynamics between various exogenous and endogenous factors revealed by our data which appeared to significantly impact the development and adoption of GM mosquitoes. Endogenous factors are related to the NPD process and characteristics of the technology itself, i.e. its biological characteristics and the scientific research process. As a living technology, it is embedded in its institutional, regulatory, cultural and socio-economic development and implementation context. The factors describe how the completion of a development stage impacts on the technology development rate with a reinforcing effect – the more successful trials that are completed, the more regulators and policy makers are aware of the technology and the greater their confidence in upcoming stages, thus reinforcing the pace of technology development. However, this may be balanced by fatigue arising from the number of trials completed to meet regulatory requirements, potentially slowing momentum. Each completed stage also influences the perception of its potential impact – positive or negative – held by the local population in the trial sites. This perception is, in turn, influenced by broader contextual factors relating to cultural and socio-economic factors and individuals’ experiences with health and healthcare (see Section 5.2).

Exogenous factors influence the pace of the NPD process through their effects on trust in and acceptance of the technology, both at the regulatory and the population levels. It emerged from the data that population acceptance was the most influential factor on the product development rate (i.e. most frequently cited in interviews and focus groups). Especially important is the population's past experience of introductions of new healthcare initiatives and technologies, as well as the way the problem being addressed is perceived, in this case malaria. Also important is a set of factors relating to the wider institutional and political context, such as the robustness of the regulatory infrastructure.

The sum of these dynamics influences both the NPD process for GM mosquito technology and its adoption on the ground, with time lags in some of the loops due to delays in system feedback (represented by the symbol II in the model diagrams). We now describe each of the sub-models and discuss the processes and relationships in more detail.

### 5.2 Sub-model 1. Institutional impact on product development process (Fig. 5)

Analysis of the interview and focus group data suggested that the product development rate was affected by the nature of the technology itself – complex and controversial – and the need for careful regulation. The institutional and political readiness of Burkina Faso to regulate the adoption of GM mosquito technology was seen by interviewees as a significant influence (see Table 1 for exemplar quotes from interviewees and focus groups). This embraces the political commitment to enforce the regulatory process, itself influenced by the preparedness of regulatory bodies in terms of their knowledge of the underlying science. Clear regulation, and the resources to enforce it, were viewed as a key part of the institutional infrastructure. Against this institutional environment there is a backdrop of pressure from groups in Burkina Faso and other countries, and the popular press. This has influenced past decisions about the adoption of GM crops, and it was expected that similar pressure would grow as the GM mosquito technology progressed towards adoption. As one interviewee put it.

“[Opposition groups] have a lot of money to spend, and a lot of effort that they can use to oppose and that's been a problem all along, especially in countries in Africa where you have well-funded opposition groups going up against these regulatory agencies. Anybody who is ideologically opposed to a genetic modification may support local people on the ground and have done frequently.” (Regulatory affairs specialist 1).

**Table 1:** Codes, themes and exemplar quotes.

2nd order themes	1st order categories	Representative codes	Quotes
GOVERNANCE	Institutional impact	Political instability	"Just the uncertainty of governments. Right now, Burkina Faso is in a good place as far as their regulatory structure not being too affected by what has happened over the last few years politically. It seems like Burkina Faso has managed to keep going despite some political upheaval, but if things should change drastically, I think that could cause a problem." Regulatory affairs specialist
		Maturity of regulatory process for GM	"The ability of the NBA (regulatory body) to conduct the assessment of the application and the evaluation of the application in a way that gives them the confidence that they've done their job properly … includes the confidence that they conducted a proper risk assessment to take the decision." Regulatory affairs specialist
		Willingness of government to enforce regulations	"The problem we have today with the implementation of the measures is the leadership at the central level. The power of the central level is very low; the DRS (*Direction Régionale de la Santé*) and MCD (*Médecin-Chef de District*) think they can do what they want regardless of the decisions of the central level. And they do so without sanctions. Unfortunately, there is virtually no sense of accountability." Founder and leader of national NGO "Even if you have a sound legislation [… you may…] have no resources to implement it." Regulatory affairs specialist
		Strength of organized opposition groups	"They have a lot of money to spend, and a lot of effort that they can use to oppose and that's been a problem all along, especially in countries in Africa where you have well-funded opposition groups going up against these regulatory agencies … Anybody who is ideologically opposed to a genetic modification may support local people on the ground and have done frequently." Regulatory affairs specialist
		Fatigue	"… if you're still trapped in the villages or still trying to get data out to those villages, then there's a sort of fatigue element that sets in and they say 'oh go away, you're never going to bring me this stuff. I don't want you here in my village anymore'.” Regulatory affairs specialist "[W]e're building up credibility and information with the committee, but they are three separate products. Normally you'd have the same product that you take through each of the steps, but … the product that you want release at the end, the gene-drive product, isn't the same as the product that you are seeing now, which is male sterile. And so what we're trying to do is to make sure they recognize elements from the male sterile that are equally applicable to gene-drive, such as the marker gene, for example." Regulatory affairs specialist
RESILIENCE, AWARENESS AND KNOWLEDGE	Understanding of own health, including malaria	Patterns of disease & health	"We have the same epidemiological profile as in other regions; above all malaria which is a public health concern." Regional director within the Ministry of Health "In most healthcare documents, malaria is listed as the leading cause of death in Burkina Faso." Project Manager within the Ministry of Health at national level
		Attitudes to malaria	"In terms of malaria, the population perceives this almost as a normal disease which is regrettable. All the protective measures that we are implementing are still not always being followed … With regards to mortality, they do not necessarily make the connection with malaria, except maybe with fetishes or sorcerers." Executive manager within the Ministry of Health
	Experience of health system and healthcare	Socio-economic resilience	"But above all, the essential, the biggest issue: poverty … As far as the Imam is concerned, people are seeing him because of a range of problems, with poverty being the biggest issue. Out of 10 people, 8 are coming because of poverty." Religious leader from a rural village "The basic problems, the priority needs are education, healthcare, sanitation, road infrastructure. Those are the main problems: health, education, roads … There you go." General Secretary of a governmental administration at regional user level
		Religious and cultural context	"Cultural acceptability counts as much as the perception that needs to be overcome." Regional director within the Ministry of Health at regional managerial level "There is no universal criterion [for acceptance]. It all depends on the communities, on innovation and on many factors that can influence and vary from one community to another. The things that are appreciated are not always the same. This depends on the range of values of the communities; there are different values between the communities." Communication officer of a national NGO at national managerial level
		Education	"Grosso modo, it's the illiteracy… people are not informed. The people do not know or don't understand what is proposed to them. In the healthcare sector, the problem is the same." Retired mayor of a mid-sized village "The Burkinabé consider [malaria] as unnatural or sometimes like a spell. Thus, [the people] invest in supernatural measures before realising that it would be enough to simply follow the health advice so that it doesn't get worse or settle in completely. This problem is part of the battle … to change the mind-set." Public Health professional within the PNLP at national policy maker level
		Access to health facilities	"There is no impetus to visit a healthcare facility if one is ill because one expects costs that could not be covered. The impetus would be to manage one's illness as good as possible which will lead to a significant delay before visiting the healthcare facilities. When the people get there, it is already too late." Communication officer of a national NGO at national managerial level
	Approaches to communicating information	Informal leaders	"The village chief has his advisors, his confidantes with whom he discusses and gives information to the population. He summons the family council and shares information. Generally, he will not be much challenged. He decides. No, no the chief will not be challenged." Retired health professional at regional level " … all innovations in the health sector involve the 'leaders of tradition', religious leaders, village chiefs and land chiefs; they pass on the information because people listen to them. The Imam and the 'leader of tradition' will transmit [the messages]. Our correspondents in the villages help us to pass on the information concerning innovation that must be introduced in the health sector." Health professional in a regional CMA "If I take a decision with the chief of the village, the entire populations follows. We organise ourselves by forming a group with the Imam and the Elders. We see what needs to be done and we take a decision to initiate what needs to be done in the neighbourhood." Chair of CVD of a rural village
		Local village system	"The village is organised based on groups and cooperatives. Essentially, there are three people with power: firstly, the CVD, then the Jeunesse (a group of young people) has a say and the Imam […] also has an incontestable power in the village." Local user level "We involve the local leaders: village chiefs, deputies, envoys, the CVD of the village, as well as the 'leader of tradition'. So people come because they participate. We need their technical help. The people say: 'there are the traditional leaders'… and then they come." Retired mayor of a mid-sized village
EVIDENCE AND TRUST	Perceived benefits and acceptance of the evidence	Legitimacy of government robustness of health system	"When a population rebels against its prefect, there is a problem: the population does not trust its prefect. Otherwise, they would have communicated. Thus, the social distance was large." Representative of a civil society group at regional level "… it is important to reform and innovate but this needs to come from the base. Until now, we have seen reforms that are coming from the top. But they implement reforms that are removed from reality. This means that we do the planning and the innovations based on the concerns of the decision makers and not based on those of the population." Founder and leader of national NGO at national managerial level
		Experience of malaria focused innovations	"When it's new people don't understand. They need to understand and generally any new activity that you introduce requires long preparations in order to sensitize the community; otherwise, your will find yourself alone and are bringing in an activity that does not work. For everything that is new you need to take the time to explain; it is necessary to start preparations early if you want to have an impact with the project. You need to be sure of this in the short-and mid-term." Member of an international NGO at international user level “[T]he suspicion is mainly cultural. [it remains] until the innovators prove it to us. We need to take into account the cultural aspects, the perception of the product, accessibility and also the support of the population." General Secretary of a network of NGOs at national technical level
		Experience of non-malaria innovations (medical and non-medical)	"In the beginning it was difficult. The women did not to vaccinate their children. But one day, the measles attacked the neighbourhood and we knew that everyone who was not vaccinated got the measles … afterwards, everyone got vaccinated. Now, all children are vaccinated." Chief of a rural village at local user level "They [the population] have habits that they are asked to change and they have difficulties adopting new behaviours. … If they don't understand this they will be reluctant. It's the level of understanding that leads to reluctance." Health professional in a regional CMA "If this [health innovation] seems easy and if you are convinced of the positive contribution to your daily practice, it works. But if it is complicated … " Manager within the drug allocation department of the Ministry of Health at national managerial level
		Familiarity and knowledge of technology	"The perception of what this technology is going to bring to you is crucial." Regulatory affairs specialist "I remember the editor of the Observer had brought up this topic at a conference. He tried to open a page for articles in Mòoré [local language]. But since the people have an 'academic logic', they wanted to build a *morophone* academy and tried to use too complicated words. At that point nobody understood anything any longer." Founder and leader of national NGO
		Familiarity and knowledge of GM technologies (agriculture and health)	"Those are new approaches to step back and find appropriate words and reduce them to an understandable language." Member of an international NGO "There is also the fact that in the communities, men are often reluctant to respond to certain interventions." Interim international consultant for Ministry of Health
		Peer pressure groups	"Some say that if doctors flattered them to get vaccinated, that shows it's because they want to inject diseases in their bodies so that they go and pay more money at the CSPS (*Centre de santé et de promotion sociale*). The doctors would get them ill so that they would go to the CSPS and pay a lot of money there. That is the idea of some of them." Member of an all-women focus group from a rural village

**Fig. 5:**
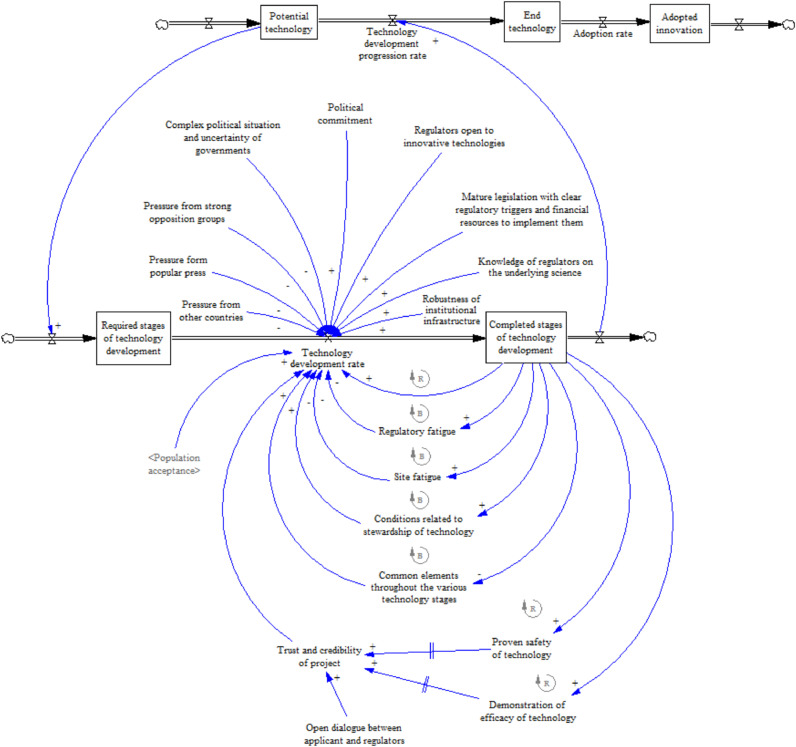
Sub-model 1. Institutional impact – policy and regulatory level.

Sub-model 1 also shows a relationship between a reinforcing loop comprising iterative learning from the R&D carried out on the project and a balancing loop representing ‘development fatigue’. The more stages of technology development that are successfully completed, the greater the evidence for safety and efficacy. This increases the project team's trust and credibility in the eyes of regulatory and policy-making bodies, as well as the population around field sites. In turn, this leads to an improved product development rate. However, data from the interviews and focus groups suggested that this process is balanced by a ‘development fatigue’ loop. As noted, development of a self-sustaining GM mosquito involves the creation of multiple strains over a series of stages. Each strain is effectively a new prototype; each stage thus has fewer common elements compared to the initial strain and the previous stage that regulatory bodies have taken a decision on. This is rather different from the product development process for more conventional health technology innovations like new drugs, described in Section 2. Each new prototype therefore requires further trials to understand the implications, but the more the required and completed stages, the higher the potential for ‘regulatory fatigue’ resulting from the expenditure of time and resources on addressing regulatory requirements. This may make it hard to keep up momentum. According to a project team member:

“The further we get along in the process, the higher the complexity, the more [regulators] may seek additional reviews, or when we get to a gene drive product … they may say ‘well let's have a socio- economic review’ … [T]hey are three separate products. Normally you'd have the same product that you take through each of the steps … but … the product that [we] want to release at the end, the gene drive product, isn't the same as the product that you are seeing now, which is male sterile. And so, what we're trying to do is to make sure they recognize elements from the male sterile that are equally applicable to gene drive.”

Comprehension of the technology at the policy level also seemed to influence the pace of development, through the number of safety reviews required for each development stage:

“What helps regulators is knowledge … whether it's from published papers or from doing the same thing in other countries, whether it is from other people doing the same thing, which helps them come to an easier decision … In Burkina you've got a ministerial sign off … it will influence regulatory if they have negative public comment.” Regulatory affairs specialist 2.

A similar effect may also emerge within the local population at trial sites. The more stages that are needed and completed without actually introducing an effective solution with tangible benefits, the more ‘site fatigue’ kicks in, again potentially slowing the product development momentum. This was described by one interviewee:

“There might be a boredom with those [engagement] conversations after a while, what we call site fatigue, especially if things get further and further delayed … If we spend ten years in a regulatory process trying to get it approved, then they'll say ‘yeah but you said it would come ten years ago, why isn't it here, … you don't know what you are doing’. Particularly if you're still … trying to get data out of those villages, then there's a sort of fatigue element that sets in and they say ‘oh go away you're never going to bring me this stuff. I don't want you here in my village anymore.’” Regulatory affairs specialist 2.

### 5.3 Sub-model 2. Impact of socio-economic and demographic context

**Fig. 6:**
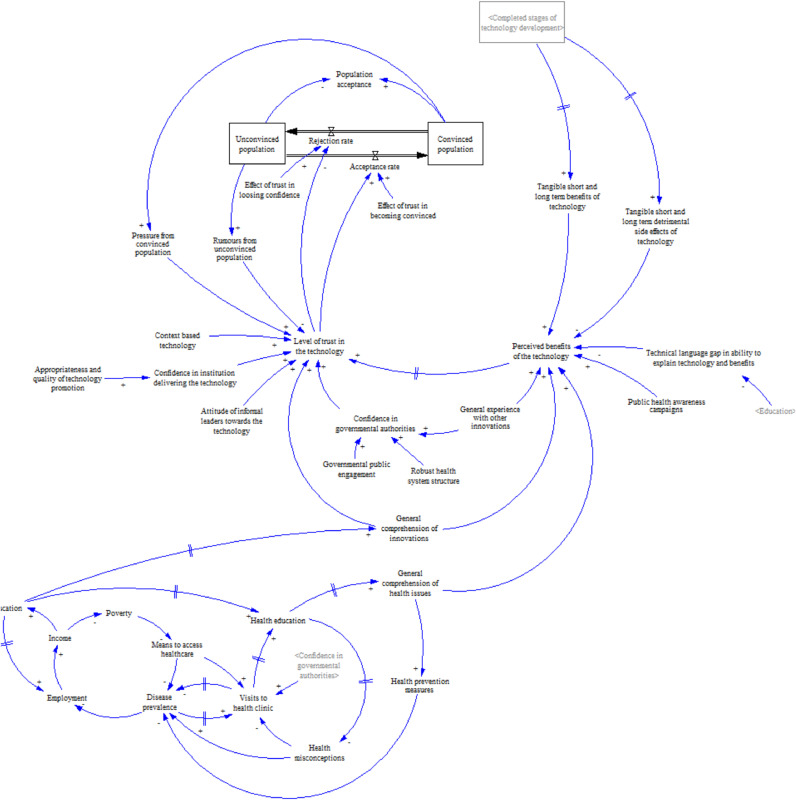
Submodel 2. Overview of the population-level acceptance loops.

Sub-model 2 conceives the overall Burkinabé population as a stock variable, subdivided into convinced and unconvinced population stocks (see Fig. 6). The rates of acceptance and rejection of the technology determine the flows between the two population stocks. The balance between the convinced and unconvinced population leads to the general level of population acceptance of the technology. Both the rate of acceptance and rejection determine the state of the wider system at any given time, i.e. progress towards the completion of the final product and its implementation. Underlying this is a set of exogenous socio-economic, cultural and demographic factors which interact with each other. Poverty, employment and access to education emerged from the interviews and focus groups as the three major issues experienced by the local population (see Table 1). The relationship between them in turn impacts on the use and experience of the health system. For example, poverty or unemployment reduce the means to access healthcare. The consequent detrimental effects on disease prevalence reinforce the inability to work. In time, however, increased disease prevalence may be balanced, as greater contact with healthcare services is encouraged and more resources are put in place.

How malaria is perceived by local populations was found to be an important influence on attitudes towards healthcare. The major health problems described by interviewees and focus groups resulted from a lack of access to primary healthcare. Malaria is widely regarded as a common disease (see Table 1). Despite campaigns to improve public understanding, there are still deeply rooted misconceptions about its transmission and symptoms are often attributed to other issues. As a result, treatment is often not sought or only after major delays:

“Every time, there are organizations that explain it. They say that the mosquito transmits malaria, every time. Up until now, the people still don't believe that. Some say that it is caused by eating mangos or fatty meals. The people don't understand that it is the mosquito that transmits malaria.” Rural village chief.

“We have always had problems because people arrive [at the facilities] too late, which means a lot of [prior] self-medication or consultations with a traditional healer.” Ministry of Health regional director.

“Traditionally, when someone had malaria, they don't care very much. If it didn't pass, they go to the traditional doctor.” Rural village religious leader.

These perceptions are reinforced by the considerable value placed on the opinions of informal leaders, peer groups and oral traditions that are ingrained in Burkinabé society (Table 1). The beliefs and views of the chief of the village, the *chef coutumier* (‘leader of traditions’), village elders, religious leaders, various local associations and village development councils may all help to reinforce misperceptions of the causes and transmission of malaria:

“Generally, in our cultures, if somebody is sick, we always think that someone has cast a spell on you … Generally, we go to the marabou or the witch doctor for a consultation rather than going straightaway to the dispensary or the hospital”. Social anthropologist working for a national Burkina Faso research institute.

A second set of exogenous factors relates to trust in health technology, and the strength of evidence for its benefits. The interviews and focus groups suggested a relationship between general comprehension of health issues and perceptions of the benefits of healthcare innovations, often expressed in relation to vaccination programmes (Table 1). Health professionals explained that better comprehension of malaria origins and transmission would lead to better understanding of the potential benefits of a technology aiming to eradicate it. A problem with GM mosquitoes, however, is that it is intrinsically hard to explain how the technology works. This leads to a high cognitive distance between technology developers, regulators and users or beneficiaries in the population, a distance further enhanced by the lack of technical words in the local languages spoken in Burkina Faso. Comprehension of the technology, including the ability of proponents to explain it in non-technical language, seemed to influence perceptions of benefits of innovations and their acceptance:

“Those are new approaches to step back and find appropriate words and reduce them to an understandable language … It's crucial to speak in a way that is accessible to the population. We didn't do this ten years ago, but we are informing the people now.” Member of an international NGO.

The degree of benefit and speed with which it is realised, as well as the ease of understanding the link between this evidence and the innovation itself, also seemed to influence the population's acceptance of healthcare innovations. Negative prior experiences translated into lower openness towards new ideas, as well as lower confidence in government authorities and NGOs. It emerged from the data that Burkinabé culture tends to favour innovations that provide immediate and tangible feedback rather than longer-term solutions. This was a problem in previous vaccine campaigns, where negative short-term side effects of vaccines played a role in rejection of the technology (see Table 1). Local populations expressed an attitude that ‘we have to see it to believe it’. As a retired health professional explained, “People need to see the advantages of the innovation. That's to say, it's a reference point.” A manager in a regional CMA (*Centres Médical avec Antenne Chirurgicale*) said, “If they have had a bad experience in one case, they will tell you by word of mouth that [vaccination] will kill. This is an element that can constitute a hurdle towards acceptance of those innovations.”

The hierarchical Burkinabé culture, emphasising peer group and social acceptance, influences the trust of individuals in official pronouncements. This enhances the competing effects of pressure from the convinced population (i.e. who believe in the benefits an innovation) and unconvinced population on acceptance or rejection of the technology:

“If you say something, the rumours start and the opposite message returns. Like for the refusal of generics [drugs]. If the doctor doesn't prescribe it, then the rumours will spread by word to mouth.” Qualified nurse at a local hospital.

“There are also rumours that are started by an individual within the population, a self-proclaimed expert, who could be semi-literate, close to a health worker or knowing how to read a little and who could distort the information. This can often be the source of the refusal of an innovation.” General Secretary of a network of NGOs.

Together, the dynamic between these factors led to a strong emphasis within the Target Malaria project on trust-building to close the cognitive distance through a carefully tailored communications and stakeholder engagement strategy. This played out both at the population and regulatory levels, as described by these interviewees:

“So, if you put in an application which you have to change and say ‘well, sorry we got that wrong’, it doesn't look like you're very trustworthy, like you've not got much credibility, because to a regulator it seems you might not know what you're doing … It's about building up a dialogue, trust, a rapport with the regulator, as well. So, they understand you, they trust you, … they trust the messenger.” Regulatory affairs specialist 1.

“When we strengthen trust, it is always good. Building trust is very important, especially if we don't politicise things.” Doctor at a regional hospital.

“We create awareness, we conduct focus groups. That's an innovation … The first step is to raise awareness to enable them to understand. Understand what the upcoming problems and the immediate solutions are, so that they are aware of the problem and asking questions about the solution.” Senior manager in a national directorate of the Ministry of Environment.

More broadly, an unwillingness to accept government pronouncements at face value may be reinforced by weaknesses in the Burkinabé health system and general scepticism towards governmental bodies due to the country's political instability. There was, however, a view that at the time of our data collection, the regulatory process for GM remained strong:

“Right now, Burkina Faso is in a good place as far as their regulatory structure not being too affected by what has happened over the last few years politically. It seems like Burkina Faso has managed to keep going despite some political upheaval, but if things should change drastically, I think that could cause a problem.” Regulatory affairs specialist 1.

In summary, the modelling shows how key components at different scales – ranging from the individual-level (e.g. beliefs held about malaria), through the population-level (e.g. local culture) to national-level components (e.g. regulatory processes) – are interlinked via a series of reinforcing and balancing feedback loops which determine the system's behaviour, potentially with time lags between the causes and effects of actions. The model therefore shows that for this technology there is a strong interaction between problem perception (the need to eradicate malaria), the characteristics of the healthcare and regulatory systems (including robustness of health services), and the local adoption system (including socio-economic and cultural factors and experience of healthcare).

## 6 Discussion

We studied a complex innovation project in real time across a complete cycle of R&D to initial implementation. Our analysis emphasized the dynamism and continuity of project events and actions, rather than states and final outcomes. In doing so we address calls in innovation and strategic management literatures for more longitudinal and implementation-focused research, and models of NPD processes that are less static, especially when the innovation is large-scale and complex (see Section 1). A project to eradicate disease-carrying mosquitoes through gene drive science provides an ideal setting for researching NPD processes in this type of innovation. The self-sustaining features of gene drive technology make it controversial; its potential impact outside the laboratory is inherently less predictable than other types of GM technology or new biopharmaceuticals. By using a system dynamics modelling approach to analyse our data, we uncovered different kinds of complexities underlying Target Malaria's innovation processes and highlight the importance of feedback loops and time lags. Some complexities in these processes arise from the need to sequence project activities across multiple system levels by aligning R&D activities, stakeholder engagement within trial sites and activities involving national regulatory bodies. Others result from the need to address the cognitive distance between scientists, policy makers and the public – gaps in understanding of the technology and its potential impacts – and time lags for corrective activities to take effect. We also observed cultural complexities which result from the contextualized settings of the sites for release of mosquitoes in the field, notably interpretations of the causes and consequences of malaria held by local populations, that in turn influence perceptions of opportunities and risks of GM mosquitoes.

Findings from our case study reinforce the view that more multifaceted and dynamic models, embracing feedback and equifinality (the idea that there can be multiple causal paths to an outcome) are needed to understand NPD processes in large scale, complex innovations.

We set out to explore NPD processes in complex innovations and their possible linearity / non-linearity, but as well as drawing conclusions on this, we believe our findings also extend literature on the relationship between product development, adoption and implementation.

### 6.1 Structured versus non-linear innovation journeys

Our findings show how characteristics of the technology in question – in this case, biological embeddedness – means that aspects of its development are highly structured by the need to address risk and safety concerns, with development proceeding linearly in a series of discrete stages. We did not identify true instances of equifinality within Target Malaria, in the sense that a desired outcome could be achieved through alternative combinations of activities (Sihvonena and Pajunen, 2019). However, our analysis suggests there are nevertheless opportunities for divergence from the prescribed NPD pathway which arise from the impact of exogenous factors – for example, popular protest against the technology - working through the system and altering its behaviour. Uncertainty over the scientific and practical success of the technology, equivocality over the evidence for its benefits, and complexity in the context for adoption all raise the possibility of combinations of endogenous and exogenous factors leading to unplanned outcomes, as much as they propel R&D towards a carefully planned stage-gate model.

As discussed in Section 2, scholars have argued that innovation journeys of large-scale, complex innovation projects involve iterative, non-linear cycles of activities (Cheng and Van de Ven, 1996; Van de Ven et al., 1999; Garud et al., 2013; Van de Ven, 2017), where the sequence of events cannot be portrayed as a “neat, step-by-step unfolding series of phases” (Anderson et al., 2004). We suggest that there are circumstances in which a structured, phased approach *can* be found within such projects, but this is partly a question of the scale at which the phenomenon is observed. In case of Target Malaria, despite the structure imposed by the regulatory and scientific process, the feedback loops and time lags we identified give its R&D and adoption system the potential to be unstable. But while rejection of the technology is scale independent because it could emerge at different levels in the system – populations in local villages, national government or global regulators or NGOs – our ability to observe the interdependencies and dynamics that lead to a particular instance of instability is influenced by the scale at which we represent the system. Our findings therefore resonate with discussions within a complexity theory tradition on the role of scale in understanding the behaviour of systems. At a coarser level of analysis – ‘the whole system’ rather than a subsystem – interdependencies are of a higher order and the pace at which their dynamics plays out is slower because higher-order interactions across multiple system levels inhibit instant feedback (Gavetti, 2005; Dattée and Barlow, 2017). Ambiguity in feedback can promote mutual confusion and flawed mental models of the system and its behaviour (Knudsen and Srikanth, 2014). Linkages between actions and outcomes become less visible and harder to interpret (Gavetti, 2005). Hence, in the case of Target Malaria the challenge for managers is to diminish the cognitive distance between project stakeholders – whether these are villagers in trial sites, project field workers, project scientists, regional administrators or national policy makers – by aligning their understandings of the technology and its evidence, and speeding-up the feedback between actions and outcomes.

### 6.2 Ambiguity between NPD, adoption and implementation

As well as lessons on the linearity of the NPD process, we also believe that the relationship between NPD, adoption and implementation is far more closely intertwined in Target Malaria than is typical in other types of innovation project. Management literature suggests that innovation adoption needs to be seen as a process, a sequence of decisions and actions, rather than a one-off, all-or-nothing event. Once a decision to adopt has been made, an innovation may need to be adapted in order to become institutionalised within an organization (Meyer and Goes, 1988; Zhu et al. 2006). It has been widely observed in healthcare that assimilation of organizational or service innovations into daily practice through a process of implementation and routinisation is required (Greenhalgh et al. 2004; May and Finch, 2009). But these literatures focus largely on implementation, post-adoption, rather than the interaction between earlier NPD stages and adoption and implementation. Clearly, the launch of an innovative product may be followed by further development or withdrawal and relaunch. There is, however, little research on this ‘pre-diffusion’ phase from the point a technology is demonstrated to the start of mainstream production and diffusion (Ortt, 2010, 2014; Tidd and Bessant, 2018).

The Target Malaria project can be seen as an example of pre-diffusion technology. Our findings suggest that the endpoint for the innovation process is unclear – when R&D ends and ‘mainstream’ adoption and diffusion begin are blurred. This is not simply a result of a regulatory process which requires a staged, iterative model of scientific research and trialling, similar to drug development. For the Target Malaria system the effects of feedback, through evidence creation and learning, at the population and regulator levels are also critical. Evidence from successfully completed trials provides confidence and reinforces the pace of NPD, but momentum may be slowed by ‘trial fatigue’, both at the regulator and population levels. In some senses, the development and introduction of gene drive mosquitoes are not unlike new drugs under an ‘adaptive licensing’ model in the pharmaceutical industry (see Section 2), in which repeated cycles of monitoring, risk mitigation and (re)licensing are an integral part of the adoption process. The difference, however, is that adaptive licensing involves targeted populations, so risks are defined and containable. The biological embeddedness of gene drive mosquitoes means the risks are potentially less containable, hence the need for increasingly large-scale and ‘real-world’ trials to build credibility, but at the risk of destabilising the NPD process through trial fatigue.

## 7 Conclusions and implications for practice and policy

The findings have two lessons for designing and managing complex NPD projects. First, it is argued that we need to reconceptualize the process of innovation, where NPD cannot be managed through a simple sequence of stages or phases (Anderson et al., 2004; Salerno et al., 2015; Van de Ven, 2017; Hullova et al., 2019a). We agree with scholars who are sceptical about the relevance of trial and error experimentation in situations where there is high uncertainty, allowing failure and learning but also containing risk (Packard et al., 2017; Gillier and Lenfle, 2019). In our case trial and error experimentation, at least in the field, is clearly undesirable because of public and regulator concern over the risks of gene drive technology. This points to a need for tight control of experimentation to control risk and avoid mistakes (Gillier and Lenfle, 2019) by balancing control and exploration (Arrighi et al., 2015). This was clearly a feature of the gene drive mosquitoes development process, where complexity was managed by adopting an approach that has overall structure but simultaneously accommodates iteration cycles (Cooper, 2016; Sommer et al., 2015; Chao et al., 2014; Salerno et al., 2015). There may be parallels here with other controversial and complex innovative technologies such as stem cell therapies, AI and nanotechnology.

Second, we highlight the need for managers to understand the complexity of the system they are situated in; the way recurring NPD activities function in a complex set of interrelationships and feedback loops, with effects that may not be immediately apparent. It is important for managers to ‘embrace the complexity’, accepting that they cannot fully control the emergent behaviour of the systems they are situated in but can control the odds of success by understanding and adapting to the system (Van de Ven et al., 1999; Van de Ven, 2017). As Sihvonena and Pajunen (2019) point out, even a rudimentary understanding of the system can help managers to better plan NPD activities. Particularly important are the identification of key stakeholders and their interdependencies with NPD processes in order to synchronize actions with the tempo of wider exogenous conditions. We therefore agree with Oyama et al. (2015) who argue that it is not the number of interdependencies, but rather their type that is important in determining NPD project outcomes.

For policy makers responsible for decisions about adoption and implementation, there are lessons which arise from the unusual properties of gene drive mosquito technology as a hybrid public and private good. The technology is a public good in the sense that decisions to adopt are made by government authorities on behalf of the entire population for their benefit, unlike insecticide treated bed nets for malaria control, where the adoption decision is made by an individual member of the population. While gene drive mosquitoes are an equitable technology because they may deliver area-wide malaria control to all, the technology is one which potentially leaves no individual freedom of choice about adoption. It cannot be compared with a vaccination programme resulting from a government decision to tackle a health problem for the benefit of all, which may still be refused by individuals, diminishing the benefits for the wider population (Luyten and Beutels, 2016). In both cases – vaccination and gene drive mosquitoes – the level of trust in the technology reinforces support for or rejection of the technology, but population acceptance appears to also have a strong impact on the product development rate because of the ‘all or nothing’ nature of adoption. A key challenge therefore is to plan for implementation of the technology while simultaneously conducting its scientific and technical development. Appropriate communications and engagement at all levels in the health and adoption systems are clearly essential, embracing not only national, regional and local institutions but also individuals in local populations. Within institutionally complex domains such as healthcare, a fruitful area for future research is understanding the different logics employed by stakeholders in innovation project and how these influence perceptions and actions.

The development and introduction of GM mosquitoes is an example of a complex innovation project, characterised by limited knowledge and distributed capabilities across the system, as well as high unpredictability and lack of clarity about cause and effect of the technology and its impact. We suggest that the use of a qualitative system dynamics model has helped to enable context, processes and outcomes of GM mosquitoes to be linked across different levels in the systems for their development and adoption. Modelling can also provide a neutral framework for encouraging dialogue between stakeholders, helping to make assumptions more explicit and provide a sounder basis for policy and practice decisions. We encourage its more widespread use.

## 8 Limitations

Most of the interviews were performed in the local language and in French, assisted by an interpreter. It is possible that ambiguity can arise from multiple meanings within the languages. Both authors have a good knowledge of French, and discussion of the emerging findings with the local Target Malaria team and translators helped to minimise ambiguity. The rigour of data collection, data analysis, and model formulation, as well as triangulation of the analysed data and the continuous discussion of the results with experts, helped to increase the internal validity of the study.

The previous employment of one author (VC) by Target Malaria raises questions of reflexivity due to her possible ‘insider’ status (Adler and Adler, 1987). While this supported data collection and access, it potentially posed challenges for objectivity (Kerstette, 2012). We addressed this by continuously questioning epistemological assumptions, experiences and possible biases through discussion with author (JB) and others both inside and outside Target Malaria. We believe that our use of multiple data sources and triangulation of data also provided assurance about the validity of the ‘story’ emerging from the data.

The technology development and adoption processes of GM mosquitoes are not only complex but are also inherently long. Although the research was conducted over a five-year period, we were only able to observe a limited part of the likely overall NPD journey. This limitation is partially mitigated through the expert interviews by asking interviewees how they envisaged the factors might change over time throughout the different technology phases and stages. However, it would be clearly beneficial to review the study findings ex-post, once the technology has been fully implemented.

## Research ethics

Ethical clearance for the study was given by Imperial College London Research Ethics Committee. The local project team also received approval via their own Institutional Research Ethics Committee in order to cover work performed for this study. Data collection followed ethical rules and principles outlined by the WHO Research Ethics Review Committee Guidelines.

## CRediT authorship contribution statement

**Valentina Cisnetto:** Conceptualization, Methodology, Investigation, Formal analysis, Writing - original draft, Writing - review & editing. **James Barlow:** Conceptualization, Methodology, Formal analysis, Writing - original draft, Writing - review & editing.

## Declaration of Competing Interest

The authors declare that they have no known competing financial interests or personal relationships that could have appeared to influence the work reported in this paper.

## Appendix Supplementary materials

Image, application 1
